# Editorial: Targeting mitochondria in aging and disease

**DOI:** 10.3389/fphar.2025.1589728

**Published:** 2025-03-21

**Authors:** Cara B. Gonzales, John M. Seubert, Antonio Marcus Paes

**Affiliations:** ^1^ Department of Comprehensive Dentistry, School of Dentistry, UT Health San Antonio, San Antonio, TX, United States; ^2^ Faculty of Pharmacy and Pharmaceutical Sciences, University of Alberta, Edmonton, AB, Canada; ^3^ Department of Physiological Sciences, Biological and Health Sciences Centre, Federal University of Maranhão, São Luís, Maranhão, Brazil

**Keywords:** mitochondria, cancer, diabetes mellitus, aging, epilepsy, abnormalities

Mitochondrial quality is increasingly recognized as an important mediator and indicator of health, aging and disease. Mitochondria are no longer just the “powerhouse of the cell” but serve multiple roles in regulating complex interactions to maintain cellular homeostasis. While mitochondrial oxidative phosphorylation (OXPHOS) has long been understood, emerging evidence over the last decade reveal mitochondrial features, activities, behaviors and functions (e.g., fission, fusion, mitophagy, apoptosis, and mitochondrial DNA; mtDNA) are critical to safeguard the cell, tissue and body from injury. Discoveries also demonstrate how mitochondria contribute to a variety of disease states including cancer, diabetes, dementia, and cardiovascular disease (CVD) (Chan, 2020; Bravo-San Pedro et al., 2017; Wang et al., 2023).

This Research Topic features 4 original articles and 3 review articles focused on the role of mitochondria in aging and disease. The major goal is to demonstrate how targeting mitochondria may provide new therapeutic routes to treat disease and combat the effects of aging thereby promoting longer, healthier lives.

The review paper “The mechanisms of action of mitochondrial targeting agents in cancer: inhibiting oxidative phosphorylation and inducing apoptosis” Yang et al., details how OXPHOS contributes to cancer cell proliferation, invasion, and migration. Circulating cancer cells, with their increased expression of complexes that drive OXPHOS and their epithelial to mesenchymal phenotype that enables metastatic spread are also discussed. Importantly, this paper details novel therapies targeting mitochondrial bioenergetics selectively in cancers thus providing new therapeutic modalities with fewer adverse effects.

Changes in mitochondrial bioenergetics and the adaptability of cancer cells to switch their metabolic profiles is demonstrated in many cancer types (Zong et al., 2016). The original research presented in “Mitochondrial energy metabolism-related gene signature as a prognostic indicator for pancreatic adenocarcinoma” Ma et al. details two prognostic mitochondrial energy metabolism-related gene signatures that accurately predicted the survival of patients with pancreatic adenocarcinoma. These may serve as therapeutic targets in future studies and possibly biomarkers of therapeutic efficacy.

Novel therapies targeting mitochondrial bioenergetics in cancers are rapidly being developed, many of which are based on naturally occurring compounds. In the original research paper “A novel benzothiazole derivative induces apoptosis via the mitochondrial intrinsic pathway producing antitumor activity in colorectal cancer” Zhou et al., a benzothiazole derivative is assessed for anticancer activity and toxicity in mouse models of colorectal cancer. Benzothiazoles (BT) are found in nature, however, BT and its derivatives are frequently synthesized for use as industrial products (e.g., fungicides), preservatives in cosmetics, antimicrobial agents, and antioxidants. Some cause toxic dermal reactions and have adverse effects on the lungs (Liao et al., 2018). In this paper, a novel BT derivative is shown to have significant anticancer activity against colorectal cancer with no clinical or histological signs of toxicity.

Plant-derived compounds are also under investigation for their anti-cancer activities. Iberverin is the primary isothiocyanate isolated from oxheart cabbage that demonstrates activity against lung cancers. The original research presented in “Iberverin exhibits antineoplastic activities against human hepatocellular carcinoma via DNA damage-mediated cell cycle arrest and mitochondrial-related apoptosis” Zhang et al. examines Iberverin effects on hepatocellular carcinoma (HCC) proliferation, migration, and apoptosis and demonstrates significant anticancer activity in mouse xenograft models of HCC.

Opposing to cell proliferation, cellular senescence, a state of irreversible cell cycle arrest, seems to contribute to age-related CVD by accumulating in cardiac muscle. Mitochondrial dysfunction plays a key role in this process, with impaired dynamics, reduced respiratory capacity, and structural changes linked to declining heart function. Targeting mitochondrial biology may help regulate senescence and improve cardiac health in aging. Epoxylipids, metabolites of polyunsaturated fatty acids, have shown protective effects by influencing mitochondrial function and reducing senescence-related damage. In the review paper “The role of CYP-sEH derived lipid mediators in regulating mitochondrial biology and cellular senescence: implications for the aging heart,” Yousef et al. potential mechanisms of how these metabolites regulate mitochondrial quality and cellular senescence are explored as new therapeutic strategies for aging-related CVD.

Diabetic ketoacidosis (DKA) is a critical complication of diabetes that arises when insulin deficiency shifts to fat as a primary energy source resulting in increased ketone production and blood acidification. Despite extensive research, the role of mitochondria in DKA remains poorly understood. Diabetes, characterized by chronic hyperglycemia and metabolic imbalances, likely interacts with mitochondrial dysfunction in complex ways. This interplay contributes to a decline in mitochondrial function, exacerbating metabolic instability, complicating treatment, and increasing risk of complications such as DKA. In “Mitochondrial proteins as therapeutic targets in diabetic ketoacidosis: evidence from Mendelian randomization analysis,” Xie et al. Mendelian randomization analysis and protein-protein interaction networks were employed to explore the relationships between mtDNA copy number and mitochondrial proteins with DKA. A causal relationship between mitochondrial function and DKA risk was identified, where increased mtDNA content and expression of specific mitochondrial proteins (MRPL32, MRPL33, COX5B, DNAJC19, NDUF88) are associated with reduced DKA risk and elevated ATP5F1B and COX4I2 correlate with increased risk. These novel findings provide insight into DKA pathogenesis and identify potential mitochondrial targets for therapeutic development.

Last but not least, the complex relationship shared by epilepsy and mitochondria, with mitochondrial dysfunction playing a crucial role in seizure susceptibility is elucidated. In the review article “Unraveling the nexus of age, epilepsy, and mitochondria: exploring the dynamics of cellular energy and excitability,” Xie et al. the authors discuss how disruptions in ATP production, calcium regulation, and oxidative stress contribute to neuronal hyperexcitability, while reactive oxygen species and neuroinflammation further drive epileptogenesis. Mitochondrial dynamics, including fusion and fission, influence seizure propagation and neuronal resilience. Notwithstanding, epigenetic modifications add another layer of complexity, shaping mitochondrial function in epilepsy. Targeted therapies such as mitochondrial antioxidants, ketogenic diets, and metabolic interventions offer promising avenues for treatment. As epilepsy prevalence rises in aging populations, personalized approaches addressing mitochondrial dysfunction may revolutionize care.

Together, these articles reinforce the pivotal role of mitochondria in aging and disease, shedding light on their impact across cancer, cardiovascular health, metabolic disorders, and neurological conditions. Beyond deepening our understanding of mitochondrial biology, these findings open exciting avenues for novel therapies aimed at restoring mitochondrial function. As research progresses, harnessing mitochondria’s potential could transform disease management, offering more precise and effective treatments. Moving forward, a deeper exploration of mitochondrial mechanisms will be key to unlocking new strategies for healthier aging and improved clinical outcomes.
